# On Vector Random Linear Network Coding in Wireless Broadcasts

**DOI:** 10.3390/e27060559

**Published:** 2025-05-26

**Authors:** Rina Su, Chengji Zhao, Qifu Sun, Zhongshan Zhang

**Affiliations:** 1 School of Cyberspace Science and Technology, Beijing Institute of Technology, Beijing 100081, China; mongolsurna@163.com; 2 School of Computer and Communication Engineering, University of Science and Technology Beijing, Beijing 100083, China; m202421003@xs.ustb.edu.cn; 3 School of Cyberspace Science and Technology, Beijing Institute of Technology (ZhuHai), Zhuhai 519088, China; zhangzs@bit.edu.cn

**Keywords:** random linear network coding (RLNC), vector linear network coding (VLNC), completion delay, wireless broadcast

## Abstract

Compared with scalar linear network coding (LNC) formulated over the finite field GF($2^{L}$), vector LNC offers enhanced flexibility in the code design by enabling linear operations over the vector space $\mathrm{GF}{\left(2\right)}^{L}$ and demonstrates a number of advantages over scalar LNC. While random LNC (RLNC) has shown significant potential to improve the completion delay performance in wireless broadcasts, most prior studies focus on scalar RLNC. In particular, it is well known that, with increasing *L*, primitive scalar RLNC over GF($2^{L}$) asymptotically achieves the optimal completion delay. However, the completion delay performance of primitive vector RLNC remains unexplored. This work aims to fill in this blank. We derive closed-form expressions for the probability distribution and the expected value of both the completion delay at a single receiver and the system completion delay. We further unveil a fundamental limitation that is different from scalar RLNC: even for large enough *L*, primitive vector RLNC over $\mathrm{GF}{\left(2\right)}^{L}$ inherently fails to reach optimal completion delay. In spite of this, the gap between the expected completion delay at a receiver and the optimal one is shown to be a constant smaller than 0.714, which implies that the expected completion delay normalized by the number *P* of original packets is asymptotically optimal with increasing *P*. We also validate our theoretical characterization through numerical simulations. Our theoretical characterization establishes primitive vector RLNC as a performance baseline for the future design of practical vector RLNC schemes with different design goals.

## 1. Introduction

Conventional scalar linear network coding (LNC) models data symbols transmitted along a network over the finite field $\mathrm{GF}(2^{L})$ and linearly combines data symbols with coding coefficients selected from $\mathrm{GF}(2^{L})$. Vector LNC [1,2,3] is an extension of conventional scalar LNC. It models the data symbols over the *L*-dimensional binary vector space $\mathrm{GF}{\left(2\right)}^{L}$ and linearly combines data packets with coding coefficients selected from L×L matrices over GF(2). Compared to scalar LNC, which provides $2^{L}$ field elements as the candidates for coding coefficient selection, a key advantage of vector LNC is that it significantly expands the number of candidates for coding coefficient selection to $2^{L^{2}}$. This expansion enhances coding flexibility. In particular, vector LNC subsumes scalar LNC in the sense that, in a single-source multicast network, every scalar LNC scheme over GF($2^{L}$) can be transformed to a vector LNC scheme over $\mathrm{GF}{\left(2\right)}^{L}$, so that the scalar scheme achieves the network’s multicast capacity if and only if the counterpart vector scheme achieves the network’s multicast capacity (see, e.g., [2,3]). In addition, Ref. [1] constructed a classical non-multicast network whose capacity can be achieved by a simple vector LNC scheme over $\mathrm{GF}{\left(2\right)}^{L}$ but cannot be achieved by scalar LNC schemes over any finite field. Various single-source multicast networks have also been constructed in [3,4,5,6] to demonstrate that the size of data symbols required by vector LNC to achieve the multicast capacity can be much smaller than that required by scalar LNC. Under the framework of vector LNC, a class of LNC schemes called circular-shift LNC was introduced in [7,8]. It models coding coefficients based on circulant matrices so as to attain lower coding complexity in comparison to scalar LNC.

Random LNC (RLNC) [9] is an important type of LNC schemes in which the coding coefficients are selected in a randomized manner. It has demonstrated significant potential to improve transmission efficiency and throughput in wireless broadcasts [8,10,11,12,13,14,15], which is a popular transmission scenario with various applications (e.g., vehicle-to-everything communication [16]). Despite the advantages of vector LNC reviewed in the previous paragraph, in the literature, most attention on the study of RLNC in wireless broadcasts is paid to scalar RLNC. In particular, it is well known that, with increasing *L*, primitive scalar RLNC over GF($2^{L}$), where “primitive” means employing independently and uniformly distributed coding coefficients from GF($2^{L}$), can achieve the optimal throughput performance. To the best of our knowledge, in the scenario of wireless broadcasts, existing research on vector RLNC has primarily focused on designing concrete vector RLNC schemes with low coding complexity [8,17]. However, for the most fundamental vector RLNC scheme, i.e.,, primitive vector RLNC over $\mathrm{GF}{\left(2\right)}^{L}$, theoretical characterization of its throughput performance remains unexplored. This work aims to fill in this theoretical blank. Similar to the consideration in [18,19,20,21,22,23,24,25], we consider systematic RLNC schemes, that is, the sender first broadcasts all original packets and then the coded packets. Following the approach in [8,19,22], we utilize *completion delay* as the core metric for evaluating throughput. This metric quantifies the total number of coded packets required by the sender until every receiver has successfully decoded all original packets.

The main contributions of this paper are summarized as follows.

- For primitive vector RLNC over $\mathrm{GF}{\left(2\right)}^{L}$, we derive closed-form characterization for the probability distribution as well as the expected value of both the completion delay at a single receiver and the system completion delay. Numerical comparison validates our theoretical characterization, demonstrating high accuracy between theoretical and simulation results, particularly for large enough *L* (Say, L≥4).
- Unlike primitive scalar RLNC over GF($2^{L}$), which is capable of asymptotically attaining optimal completion delay when *L* increases, we reveal that, even for large enough *L*, primitive vector RLNC over $\mathrm{GF}{\left(2\right)}^{L}$ fails to reach optimal completion delay, but the gap between the expected completion delay at a receiver and the optimal one is shown to be a constant smaller than 0.714.
- We reveal that, for primitive vector RLNC over $\mathrm{GF}{\left(2\right)}^{L}$, the normalized expected completion delay per original packet asymptotically converges to its optimal value as *P* grows.

Our theoretical characterization on the completion delay performance of primitive vector RLNC provides a theoretical benchmark for the future design of vector RLNC schemes with different design goals.

The rest of this paper is structured as follows. Section 2 establishes the system model and reviews known results on perfect RLNC, a class of RLNC schemes that attain the optimal completion delay performance. Section 3 theoretically characterizes the probability distribution and the expected value of completion delay of primitive vector RLNC over $\mathrm{GF}{\left(2\right)}^{L}$. The theoretical characterization is numerically compared with simulation results in Section 4. The paper is concluded in Section 5.

## 2. Preliminaries

**Figure 1:**
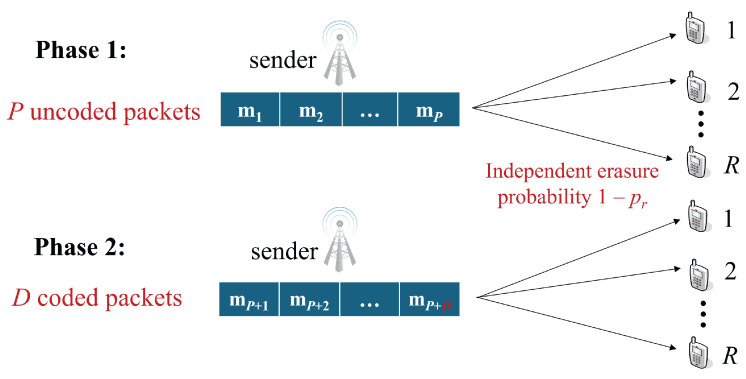
System model of a wireless broadcast network, consisting of 1 sender and *R* receivers.

As illustrated in Figure 1, we consider a single-hop wireless broadcast network without feedback, where a single sender aims to deliver a total of *P* original packets to *R* receivers. Each packet contains *M* bits. During each timeslot, the sender is allowed to transmit one packet, which is received by all receivers. The communication link between the sender and every receiver is modeled as an independent memoryless erasure channel, where receiver *r* experiences a packet erasure probability of $1-p_{r}$. The goal for every receiver is to successfully recover all *P* original packets.

In this paper, all RLNC transmission strategies adopt a *systematic* structure, which has also been considered in [18,22]. Specifically, during the initial transmission phase, the sender sequentially broadcasts all *P* original packets $m_{1},m_{2},\ldots ,m_{P}$. Following this, in the second phase the sender transmits coded packets, each formed as a linear combination of the *P* original packets. This process continues until all receivers successfully recover the entire set of *P* original packets. The term *completion delay* refers to the number of coded packets sent during this second phase. As emphasized in prior studies on RLNC performance in wireless broadcast settings [8,19,22], completion delay serves as a fundamental indicator of transmission efficiency. Non-systematic codes usually incur higher completion delay than systematic codes [19]. This is due to the fact that the first *P* transmitted packets are not original packets, but randomly coded packets, which are *not* necessarily linearly independent.

The conventional scalar RLNC scheme operates over the finite field GF($2^{L}$), where every packet is treated as a row vector composed of $\frac{M}{L}$ symbols belonging to GF($2^{L}$) (To simplify the exposition, we assume *L* divides *M*. In practical systems, since M≫L, padding with dummy bits ensures this condition is met). In this scheme, every packet transmitted by the sender is constructed as a GF($2^{L}$)-linear combination of the *P* original packets $m_{1},m_{2},\ldots ,m_{P}$. In particular, during the second transmission phase, each coded packet $m_{P+d}$ (for d≥1) generated by the sender can be represented as

(1) $$m_{P+d}=\sum_{j=1}^{P}\gamma_{j}m_{j},$$

where each coding coefficient $\gamma_{j}$ is uniformly and independently drawn from GF($2^{L}$).

To enable receivers to interpret how $m_{P+d}$ is generated from the original packets, a *global encoding kernel* $f_{P+d}={\left[\gamma_{1},\ldots ,\gamma_{P}\right]}^{T}$ is appended to the packet as a header. For the *P* original packets, their corresponding global encoding kernels form the P×P identity matrix, i.e., ${\left[f_{j}\right]}_{1\leq j\leq P}=I_{P}$. Once a receiver *r* successfully receives *P* packets with linearly independent global encoding kernels, it can recover the entire *P* original packets through decoding.

The RLNC schemes we consider in this paper are *vector RLNC* schemes, which are defined over GF${\left(2\right)}^{L}$. Each packet $m_{j}$ of *M* bits is interpreted as a row vector $\left[s_{j,1},s_{j,2},\ldots ,s_{j,\frac{M}{L}}\right]$ comprising $\frac{M}{L}$ symbols, where each symbol $s_{j,j^{\prime }}$ is treated as an *L*-bit row vector defined over GF(2). For an L×L matrix K over GF(2), the linear operation $K\circ m_{j}$ is defined symbol-wise as

(2) $$K\circ m_{j}=\left[s_{j,1}K,s_{j,2}K,\ldots ,s_{j,\frac{M}{L}}K\right].$$

Hence, for every d≥1, the coded packet $m_{P+d}$ that is randomly produced by the sender during the second phase can be represented as

(3) $$m_{P+d}=\sum_{j=1}^{P}K_{j}\circ m_{j},$$

where the coding coefficients $K_{j}$ are randomly and independently selected from L×L matrix over GF(2). We define the *global encoding kernel* of a packet as a PL×L matrix over GF(2). Every global encoding kernel can be viewed as a P×1 block matrix, where each block corresponds to a coding coefficient represented by an L×L matrix over GF(2). For an original packet $m_{j}$, 1≤j≤P, its global encoding kernel $F_{j}$ contains the identity matrix $I_{L}$ in the $j^{\mathrm{th}}$ block entry and L×L zero matrices in all other positions. Consequently, ${\left[F_{j}\right]}_{1\leq j\leq P}=I_{PL}$. For a coded packet $m_{P+d}=\sum_{j=1}^{P}K_{j}\circ m_{j}$, its global encoding kernel $F_{P+d}$ is defined as $F_{P+d}={\left[K_{1}^{T}\;K_{2}^{T}\;\ldots \;K_{P}^{T}\right]}^{T}$. For vector codes, a receiver can decode the *P* original packets upon receiving any *P* packets whose global encoding kernels are linearly independent (i.e., their concatenation has full rank PL).

*Perfect* RLNC [8,22] is a class of RLNC scheme where the encoded packets generated by the source node exhibit the strongest possible linear independence. In particular, a receiver is able to recover all *P* original packets upon successfully receiving arbitrary *P* perfect RLNC packets. Therefore, perfect RLNC is optimal in terms of completion delay, so it serves as an important benchmark scheme in the literature of RLNC in wireless broadcasts. For perfect RLNC, let $D_{r}^{\mathrm{perf}}$ and $D^{\mathrm{perf}}=\max_{1\leq r\leq R}D_{r}^{\mathrm{perf}}$ respectively denote the completion delay at single receiver *r* and the system completion delay. It is known that $D_{r}^{\mathrm{perf}}$ follows the negative binomial distribution with parameter *P* and $p_{r}$ [8]. Consequently, the distribution of $D_{r}^{\mathrm{perf}}$ follows

(4) $$\mathrm{Pr}\left(D_{r}^{\mathrm{perf}}\leq d\right)=I_{p_{r}}\left(P,d+1\right),$$

where $I_{p_{r}}\left(P,d+1\right)$ is the regularized incomplete beta function and is expressed as

(5) Ipr(P,d+1)=∑j=0dP+j−1P−1prP(1−pr)j.

Meanwhile,

(6) $$E\left[D_{r}^{\mathrm{perf}}\right]=\frac{P}{p_{r}}-P.$$

Based on Equation (4), we further have

(7) $$\mathrm{Pr}\left(D^{\mathrm{perf}}\leq d\right)=\prod_{1\leq r\leq R}I_{p_{r}}\left(P,d+1\right),E\left[D^{\mathrm{perf}}\right]=\sum_{d\geq 0}\left(1-\prod_{1\leq r\leq R}I_{p_{r}}\left(P,d+1\right)\right).$$

## 3. Theoretical Analyses of Vector RLNC

Analogous to conventional scalar RLNC over GF($2^{L}$), in which coding coefficients are independently and uniformly selected from GF($2^{L}$), the most fundamental vector RLNC scheme selects coding coefficients independently and uniformly from all L×L matrices over GF(2). Unless otherwise specified, such an RLNC scheme is referred to as *primitive* vector RLNC over $\mathrm{GF}{\left(2\right)}^{L}$. To the best of our knowledge, the completion delay performance of primitive vector RLNC has not been theoretically analyzed before. This section aims to address and fill in this blank.

For primitive vector RLNC over $\mathrm{GF}{\left(2\right)}^{L}$, let $D_{r}$ and $D=\max_{1\leq r\leq R}D_{r}$ respectively denote the completion delay at single receiver *r* and the system completion delay. The analysis of $D_{r}$ requires the following lemma. For d≥0, let $R_{PL\times (P+d)L}$ be a randomly generated matrix over GF(2), in which every entry is independently and uniformly distributed over {0,1}. Let $q_{d}$ represent the full rank probability of $R_{PL\times (P+d)L}$.

**Lemma** **1.** *The full rank probability of $R_{PL\times (P+d)L}$ is given by*

(8) $$q_{d}=\mathrm{Pr}\left\{\mathrm{rank}\left(R_{PL\times (P+d)L}\right)=PL\right\}=\prod_{l=0}^{PL-1}\left(1-2^{-((P+d)L-l)}\right).$$

*In particular, when d=0,*

$$0.2887<q_{0}\leq 1/2.$$

**Proof.**  Let $r_{l}$ denote the $l^{\mathrm{th}}$ row of the binary matrix $R_{PL\times (P+d)L}$. Assume that we build the random matrix $R_{PL\times (P+d)L}$ row by row. The probability for $r_{0}$ to be full-rank is $1-\frac{1}{2^{(P+d)L}}$. Under the assumption that the first l rows $r_{0},\ldots ,r_{l-1}$ are linearly independent, as there are $2^{l}$ different GF(2)-linear combinations of $r_{0},\ldots ,r_{l-1}$, the probability of the $l^{\mathrm{th}}$ row $r_{l}$ being linearly independent of all previous l rows is $1-\frac{2^{l}}{2^{(P+d)L}}=1-2^{-((P+d)L-l)}$. Hence,

(9) $$\begin{array}{cc} \; & \mathrm{Pr}\{\mathrm{rank}\left(R_{PL\times (P+d)L}\right)=PL\}\\ = & \prod_{l=0}^{PL-1}\mathrm{Pr}\left\{r_{l}\;\mathrm{linearly}\;\mathrm{independent}\;\mathrm{of}\;r_{0},\ldots ,r_{l-1}|r_{0},\ldots ,r_{l-1}\mathrm{linearly}\;\mathrm{independent}\right\}\\ = & \prod_{l=0}^{PL-1}\left(1-2^{-((P+d)L-l)}\right). \end{array}$$

When d=0, the size of matrix $R_{PL\times (P+d)L}$ becomes PL×PL, and

(10) $$q_{0}=\prod_{l=0}^{PL-1}\left(1-2^{-(PL-l)}\right)=\prod_{k=1}^{PL}\left(1-2^{-k}\right).$$

When PL increases, $q_{0}$ decreases. Thus, when PL=1, $q_{0}$ is maximized and equal to 1/2. It can also be readily checked that as PL increases, $q_{0}$ converges to 0.2887. Thus, $0.2887<q_{0}\leq 1/2$. □

For scalar RLNC defined over GF($2^{L}$), it has been widely recognized (see, e.g., [8]) that, as *L* grows, the expected completion delay asymptotically approaches the optimal value $E[D^{\mathrm{perf}}]$, i.e., under the assumption of perfect RLNC.

On the contrary, as a consequence of Lemma 1, it turns out that a similar conclusion cannot be drawn for primitive vector RLNC over $\mathrm{GF}{\left(2\right)}^{L}$ regardless of the choice of *L*.

**Proposition** **1.** *For primitive vector RLNC over $\mathrm{GF}{\left(2\right)}^{L}$, $E\left[D\right]\neq E\left[D^{\mathrm{perf}}\right]$ for any choice of L.*

**Proof.**  Perfect RLNC assumes that receiver *r* is able to recover the original *P* packets upon successfully receiving any *P* packets. If this condition holds for primitive RLNC over $\mathrm{GF}{\left(2\right)}^{L}$, then, for any *P* packets successfully received by *r*, the corresponding *P* global encoding kernels each of size PL×L, when concatenated column-wise, can form a full-rank PL×PL matrix over GF(2), say $R_{PL\times PL}$. Among the *P* received packets, it is assumed that *N* are coded packets. As the global encoding kernels for original packets consist of unit column vectors, the PL×PL full-rank matrix $R_{PL\times PL}$ can be reduced to an NL×NL full-rank matrix $R_{NL\times NL}$, in which every entry is randomly selected from GF(2). However, according to Lemma 1, for any *L* there is a nonzero probability that $R_{PL\times PL}$ is not full rank, so that a contradiction is drawn. Hence, the perfect RLNC assumption does not hold for primitive vector RLNC for any *L*. □

Even though primitive vector RLNC over $G{\left(2\right)}^{L}$ cannot asymptotically achieve the optimal expected completion delay with increasing *L*, based on the following characterization of the distribution of completion delay, we assert that primitive vector RLNC over $G{\left(2\right)}^{L}$ asymptotically achieves the optimal expected completion delay with increasing *P*.

Throughout our theoretical analysis, we make the assumption that *L* is large enough (say, L≥8). The main reason for this is twofold. This assumption is motivated by two crucial considerations. First, it guarantees that the full-rank probability $q_{2}$ is equal to 1 for all values of *P*, as established in Equation (8), thereby significantly simplifying our derivation of the completion delay distribution. Second, it ensures that the probabilities $q_{0}$ and $q_{1}$ remain effectively constant across different *P*, which is essential for maintaining the accuracy of our analytical results. For instance, when L=8, $q_{0}=0.2888$ for P≥1 and $q_{0}=0.2899$ for P=1, and $q_{1}=0.9961$ for all P≥1.

**Theorem** **1.** *For primitive vector RLNC over $\mathrm{GF}{\left(2\right)}^{L}$, the distribution of completion delay $D_{r}$ at single receiver r is characterized as $\mathrm{Pr}\left\{D_{r}=0\right\}=p_{r}^{P}$, and for d≥1,*

(11) $$\begin{array}{cc} \mathrm{Pr}\{D_{r}\leq d\}= & p_{r}^{P}+\left(I_{p_{r}}\left(P,d+1\right)-p_{r}^{P}\right)q_{0}+\\ \; & \left(I_{p_{r}}\left(P+1,d\right)-p_{r}^{P}I_{p_{r}}\left(1,d\right)\right)\left(q_{1}-q_{0}\right)+\\ \; & \left(I_{p_{r}}\left(P+2,d-1\right)-p_{r}^{P}I_{p_{r}}\left(2,d-1\right)\right)\left(q_{2}-q_{1}\right) \end{array}$$

**Proof.**  The technical proof is given in Appendix A. □

**Theorem** **2.** *For primitive vector RLNC over $\mathrm{GF}{\left(2\right)}^{L}$, the expected completion delay at a single receiver is given by*

(12) $$E\left[D_{r}\right]=\frac{P}{p_{r}}-P+\frac{1-p_{r}^{P}}{p_{r}}\left(2-q_{0}-q_{1}\right).$$

**Proof.**  The technical proof of Equation (12) based on Equation (11) is given in Appendix B. □

In addition to the technical derivation of $E[D_{r}]$ in Appendix B, based on the distribution of $D_{r}$, we can also analytically characterize $E[D_{r}]$ based on the concept of negative binomial distribution and the full rank probability of $q_{0}$, $q_{1}$, $q_{2}$ as follows.

First, it takes an average of $\frac{P}{p_{r}}-P$ transmissions until receiver *r* successfully receives *P* packets. Assume among these *P* successfully received packets, U(≤P) are original packets received in the first transmission phase and P−U are coded packets received at the second transmission phase. Assume the case P−U>0, which happens with probability $1-p_{r}^{P}$. In this case, receiver *r* is able to utilize P−U received coded packets to recover P−U missing original packets with probability $q_{0}$. Thus, with probability $1-q_{0}$, receiver *r* needs to receive at least one more coded packet, after an average of $1/p_{r}$ transmissions. Upon receiving P−U+1 coded packets, the probability of receiver *r* to recover P−U missing original packets is $q_{1}$. Consequently, with probability $1-q_{1}$, receiver *r* needs to receive an extra coded packet, after an average of $1/p_{r}$ transmissions. Upon receiving P−U+2 coded packets, the probability of receiver *r* to recover P−U missing original packets is $q_{2}$, which is equal to 1 under the assumption that *L* is large enough. To sum up, the expected completion delay at receiver *r* can be characterized as

(13) $$E\left[D_{r}\right]=\frac{P}{p_{r}}-P+\left(1-p_{r}^{P}\right)\left(\frac{1}{p_{r}}\left(1-q_{0}\right)+\frac{1}{p_{r}}\left(1-q_{1}\right)\right).$$

It can be readily checked that Equation (13) and Equation (12) are mathematically equivalent representations of the expected completion delay $E[D_{r}]$.

**Remark** **1.** *Under the assumption that L is large enough, our theoretical characterization of the expected completion delay for primitive vector RLNC over $\mathrm{GF}{\left(2\right)}^{L}$ is invariant to L. This invariance results in a fixed gap $\frac{1-p_{r}^{P}}{p_{r}}\left(2-q_{0}-q_{1}\right)$ in completion delay between primitive vector RLNC and perfect RLNC, even when L increases to infinity. Despite this, the next corollary asserts that the expected completion delay normalized by P asymptotically approaches the optimal value with increasing P.*

**Corollary** **1.** *For primitive vector RLNC over $\mathrm{GF}{\left(2\right)}^{L}$ (for large enough L), $E[D_{r}]$ is upper bounded by*

(14) $$\begin{array}{c} E\left[D_{r}\right]\leq \frac{1}{p_{r}}\left(P+0.714\right)-P. \end{array}$$

*Consequently, with increasing P,*

(15) $$\lim_{P\to \infty }\frac{E[D_{r}]}{P}=\lim_{P\to \infty }\frac{E[D_{r}^{\mathrm{perf}}]}{P}=\frac{1}{p_{r}}-1.$$

**Proof.**  According to Equation (12), $E[D_{r}]$ is upper bounded by $\frac{1}{p_{r}}\left(P+2-q_{0}-q_{1}\right)-P$. Since we assume L is large enough, $q_{2}=1,q_{1}=0.9961,q_{0}=0.2899$. We thus have $\frac{1}{p_{r}}\left(P+2-0.9961-0.2899\right)-P=\frac{1}{p_{r}}\left(P+0.714\right)-P$, which is the upper bound of $E[D_{r}]$ for primitive vector RLNC over $\mathrm{GF}{\left(2\right)}^{L}$. Equation (15) is a direct consequence of (14). □

**Remark** **2.** *The corollary above asserts that, for primitive vector RLNC over $\mathrm{GF}{\left(2\right)}^{L}$ with large enough L, regardless of the packet erasure probability $1-p_{r}$, a receiver only needs to successfully receive P+0.714 packets on average to recover all P original packets, where 0.714 is derived from $2-q_{0}-q_{1}$. In comparison, it has been proven that, for RLNC over GF(2) (which is equivalent to primitive vector RLNC over GF(2)), a receiver needs to successfully receive at most P+2 packets on average to recover all P original packets [26]. This signifies that the assumption of larger L allows us to deduce a tighter upper bound on $E[D_{r}]$.*

**Proposition** **2.** *For vector RLNC over $\mathrm{GF}{\left(2\right)}^{L}$, the distribution of system completion delay D is given by*

(16) $$\mathrm{Pr}\left(D\leq d\right)=\prod_{1\leq r\leq R}\mathrm{Pr}\left(D_{r}\leq d\right)$$

*where $\mathrm{Pr}(D_{r}\leq d)$ is explicitly characterized in Theorem 1. Based on the distribution of D, the expected system completion delay D can be characterized as*

(17) $$E\left[D\right]=\sum_{d\geq 0}\mathrm{Pr}\left(D>d\right)=\sum_{d\geq 0}\left(1-\prod_{1\leq r\leq R}\mathrm{Pr}\left(D_{r}\leq d\right)\right).$$

This section theoretically analyzed the distribution as well as the expectation of completion delay for primitive vector RLNC over $\mathrm{GF}{\left(2\right)}^{L}$, which serves as a benchmark scheme for the future study of vector RLNC.

## 4. Numerical Validation

In this section, we present the numerical results of primitive vector RLNC schemes over $\mathrm{GF}{\left(2\right)}^{L}$, with *L* respectively set as 1,4,8,10, and compare the simulation results to the theoretical derivations obtained in the previous section. The erasure probability at each receiver is fixed at 0.2. The completion delay performance depicted in this section is normalized by the packet number *P*. In the figure legend, theoretical results are labeled as “theo” and simulation results are labeled as “simu”.

Recall that, in the previous section, the theoretical analysis is based on the assumption that *L* is large enough so that $q_{2}$, defined in Equation (8), is always equal to 1 and the value of $q_{0}$ and of $q_{1}$ stays approximately the same, regardless of the choice of *P*. In this section, when L=8,10, we directly adopt Equation (11) in Theorem 1 to calculate the distribution of $D_{r}$. When L=1, $q_{2}$ cannot be assumed to be 1 anymore; as a direct extension of (11), we adopt the following equation to calculate the distribution of $D_{r}$ to make the theoretical characterization more accurate:

(18) $$\begin{array}{cc} \mathrm{Pr}\{D_{r}\leq d\}= & p_{r}^{P}+\left(I_{p_{r}}\left(P,d+1\right)-p_{r}^{P}\right)q_{0}+\\ \; & \sum_{j\geq 1}I_{p_{r}}\left(P+j,d-j+1\right)-p_{r}^{P}I_{p_{r}}\left(j,d-j+1\right))\left(q_{j}-q_{j-1}\right), \end{array}$$

and $E[D_{r}]$ and E[D] are respectively calculated based on $\sum_{d\geq 0}\mathrm{Pr}\left(D_{r}>d\right)$ and Equation (17).

**Figure 2:**
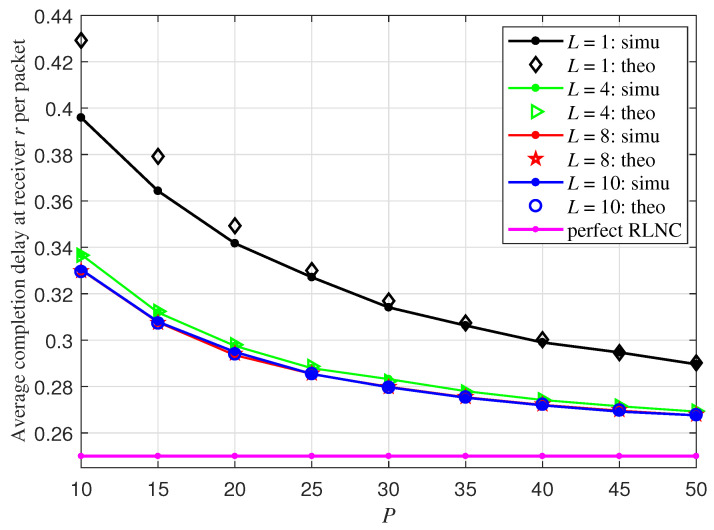
Average completion delay per packet at receiver *r* of different primitive RLNC schemes over $\mathrm{GF}{\left(2\right)}^{L}$ with R=1, $p_{r}=0.8$, and various *P*.

Figure 2 plots the average completion delay per packet at receiver *r* based on the theoretical and simulation results of primitive vector RLNC schemes over different $\mathrm{GF}{\left(2\right)}^{L}$. The expected completion delay $1/p_{r}-1=0.25$ at receiver *r* of perfect RLNC is also depicted for comparison. From this figure, the following observations can be made. First, the average completion delay at receiver *r* of every primitive vector RLNC scheme decreases with increasing *P*. Second, when L=4,8,10, there is no visually distinguishable difference between the theoretical values of $E[D_{r}]$ characterized in Equation (12) and the numerical results. This validates the correctness of our theoretical derivation in Theorem 1 based on the assumption of large enough *L*. In contrast, when L=1 and P≤40, the theoretical value of $E[D_{r}]$ in Equation (12) is higher than the actual simulation value. This is because, without the assumption that *L* is large enough, the theoretical value $q_{d}$ we adopted in Theorem 1 is smaller than the actual probability of a random $R_{(P-U)L\times (P-U+d)L}$ matrix being full-rank, which leads to the distribution $\mathrm{Pr}\{D_{r}\leq d\}$ characterized in Equation (18) smaller than the actual one. However, with increasing *P*, the theoretical value coincides with the simulation one. Furthermore, Figure 2 reveals that, when L≥8, the increase of *L* will no longer lower the expected completion delay $E[D_{r}]$ of primitive vector RLNC over $\mathrm{GF}{\left(2\right)}^{L}$, while there is a noticeable gap between $E[D_{r}]$ and $E\left[D_{r}^{\mathrm{perf}}\right]=\frac{1}{p_{r}}-1=0.25$ of perfect RLNC. This observation is in line with the discussion in Remark 1. According to Equation (12), when *P* continues to increase, $E[D_{r}]$ converges asymptotically to 0.25, and thus has the asymptotical optimal completion delay performance. This finding aligns with the conclusion of (15) in Corollary 1. Last, Table 1 illustrates that, when L=8, the expected completion delay per packet at single receiver *r* is lower than the theoretical upper bound computed via Equation (14), thereby validating the accuracy of the upper bound in Equation (14).

**Figure 3:**
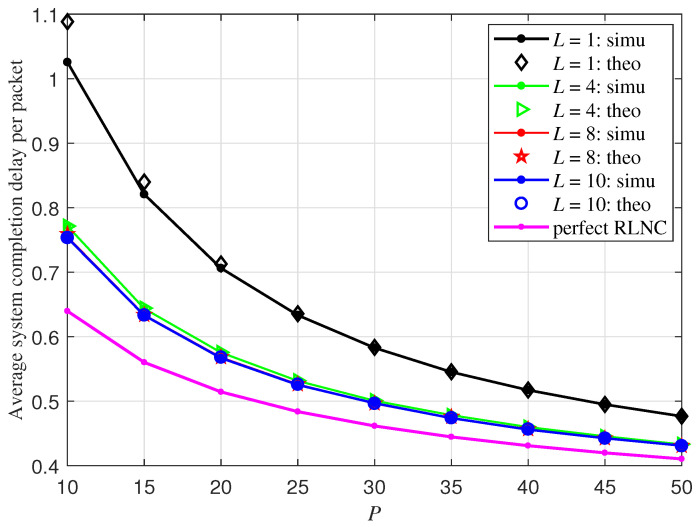
Average system completion delay per packet of different primitive RLNC schemes over $\mathrm{GF}{\left(2\right)}^{L}$ with R=20, $p_{r}=0.8$ and various *P*.

Theoretical and simulation results for the average system completion delay of primitive vector RLNC schemes over $\mathrm{GF}{\left(2\right)}^{L}$ are compared in Figure 3 under different parameter settings. The number of receivers is set to be 20. As observed from Figure 3, the performance gap in completion delay among primitive vector RLNC schemes with different *L* diminishes as *P* increases. Moreover, when L≥8, the increase of *L* will not lower the expected completion delay E[D] of primitive vector RLNC over $\mathrm{GF}{\left(2\right)}^{L}$ any more. Furthermore, when *P* continues growing, all primitive vector RLNC schemes asymptotically approach the performance of perfect RLNC. The same as the observation of Figure 2 and Figure 3 demonstrates that, when L≥4, there is no visually distinguishable difference between the theoretical characterization of E[D] in Proposition 2 and the simulation results. For the case L=1, the theoretical characterization of E[D] becomes more accurate as *P* increases, because E[D] is deduced based on the distribution of $D_{r}$ in Equation (18), which is smaller than the actual value but converges to the actual one with increasing *P*.

## 5. Conclusions

In the context of primitive vector RLNC over $\mathrm{GF}{\left(2\right)}^{L}$, we present closed-form expressions for both the probability distribution and the expected value of the individual completion delay $D_{r}$ at receiver *r*, as well as the overall system completion delay *D*, providing exact analytical tools for throughput performance evaluation. Even for large enough *L*, primitive vector RLNC over $\mathrm{GF}{\left(2\right)}^{L}$ inherently fails to reach optimal completion delay, but the gap between $E[D_{r}]$ and the optimal one is shown to be a constant smaller than 0.714, which implies that $E[D_{r}]/P$ is asymptotically optimal with the increasing number *P* of original packets. Numerical simulations validate our theoretical derivations, demonstrating near-perfect alignment between theoretical and empirical results. Our findings on the completion delay performance of primitive vector RLNC provide a theoretical benchmark for the future design of practical vector RLNC schemes with different design goals.

## Figures and Tables

**Table 1:** Comparison between simulation results and the derived upper bound Equation (14) for different values of *P*.

*P*	10	15	20	25	30	35	40
simu	0.3302	0.3077	0.2935	0.2854	0.2799	0.2754	0.2721
Equation (14)	0.3393	0.3095	0.2946	0.2857	0.2799	0.2755	0.2723

## Data Availability

The theoretical analysis and key numerical results are fully presented in this paper. Raw simulation data and code are available from the corresponding author upon reasonable request.

## Appendix A. Proof of Theorem 1

Among the *P* original packets sent by the sender in the first phase of transmission, let *U* denote the number of successfully received ones by receiver *r*. Let *V* denote the total number of packets that need to be successfully received by *r* in order to recover the *P* original packets. If U=P, then V=P, too. If U<P, then *V* is possibly larger than *P*, because the column-wise juxtaposition of the global encoding kernels for the first *P* successfully received packets is not necessarily full rank. However, based on the assumption of large enough *L*, so that $q_{2}=1$, we have V≤P+2.

For a positive integer P≥1, denote by $N_{P}$ the negative binomial random variable with parameters *P* and $p_{r}$.

It is obvious that $\mathrm{Pr}\left\{D_{r}=0\right\}=\mathrm{Pr}\left\{U=P\right\}=p_{r}^{P}$. For d≥1, the event $D_{r}\leq d$ occurs if and only if one of the following four disjoint cases occurs.

- Case *A*: U=P and thus V=P, which has probability $p_{r}^{P}$ to occur.
- Case *B*: $N_{P}\leq d$, U<P, and V=P. The probability of this case to occur is analyzed as follows. First,
  (A1) $$\mathrm{Pr}\left\{N_{P}\leq d,U<P\right\}=\mathrm{Pr}\left\{N_{P}\leq d\right\}-\mathrm{Pr}\left\{U=P\right\}=I_{p_{r}}\left(P,d+1\right)-p_{r}^{P}.$$
  Under the conditions $N_{P}\leq d$ and U<P, the probability of V=P is equal to the probability that the column-wise juxtaposition of the global encoding kernels for the V=P successfully received packets, which is a PL×PL matrix denoted by $R_{PL\times PL}$ over GF(2), is full rank. As the global encoding kernels for original packets consist of unit column vectors, $R_{PL\times PL}$ can be reduced to an (V−U)L×(V−U)L matrix $R_{(V-U)L\times (V-U)L}$ in which every entry is independently and randomly selected from GF(2), so that $\det\left(R_{PL\times PL}\right)=\det\left(R_{(V-U)L\times (V-U)L}\right)$. Based on the assumption that *L* is large enough, the probability for $R_{(V-U)L\times (V-U)L}$ to be full rank is $q_{0}$, regardless of the value of V−U>1. Thus,
  (A2) $$\mathrm{Pr}\left\{V=P|N_{P}\leq d,U<P\right\}=q_{0}$$
  and
  (A3) $$\mathrm{Pr}\left\{\mathrm{Case}\;B\;\mathrm{occurs}\right\}=\left(I_{p_{r}}\left(P,d+1\right)-p_{r}^{P}\right)q_{0}.$$
- Case *C*: $N_{P+1}\leq d-1$, U<P, and V=P+1. First,
  (A4) $$\begin{array}{cc} \mathrm{Pr}\{N_{P+1}\leq d-1,U<P\} & =\mathrm{Pr}\left\{N_{P+1}\leq d-1\right\}-\mathrm{Pr}\left\{N_{P+1}\leq d-1,U=P\right\}\\ \; & =I_{p_{r}}\left(P+1,d\right)-p_{r}^{P}I_{p_{r}}\left(1,d\right), \end{array}$$
  where the last equality holds due to $\mathrm{Pr}\left\{N_{P+1}\leq d-1|U=P\right\}=\mathrm{Pr}\left\{N_{1}\leq d-1\right\}=I_{p_{r}}\left(1,d\right)$. In a similar argument to that in Case *B*, under the conditions $N_{P+1}\leq d-1$ and U<P, the probability of V=P+1 is equal to the probability that a randomly generated (V−U)L×(V−U+1)L matrix $R_{(V-U)L\times (V-U+1)L}$ has full rank, while its first (V−U)L columns are not linearly independent. This probability is equal to $q_{1}-q_{0}$. Consequently,
  (A5) $$\mathrm{Pr}\left\{\mathrm{Case}\;C\;\mathrm{occurs}\right\}=\left(I_{p_{r}}\left(P+1,d\right)-p_{r}^{P}I_{p_{r}}\left(1,d\right)\right)\left(q_{1}-q_{0}\right).$$
- Case *D*: $N_{P+2}\leq d-2$, U<P, and V=P+2. In an essentially same argument as that for Case *C*, we have
  (A6) $$\mathrm{Pr}\left\{\mathrm{Case}\;D\;\mathrm{occurs}\right\}=\left(I_{p_{r}}\left(P+2,d-1\right)-p_{r}^{P}I_{p_{r}}\left(2,d-1\right)\right)\left(q_{2}-q_{1}\right).$$

Equation (11) can now be proven to be correct by adding (A3), (A5), (A6), and $\mathrm{Pr}\left\{\mathrm{Case}\;A\;\mathrm{occurs}\right\}=p_{r}^{P}$ up.

## Appendix B. Proof of Theorem 2

We first express $E[D_{r}]$ in the following way,

(A7) $$E\left[D_{r}\right]=\sum_{d\geq 0}\mathrm{Pr}\left\{D_{r}>d\right\}=1-p_{r}^{P}+\sum_{d\geq 1}\left(1-\mathrm{Pr}\left\{D_{r}\leq d\right\}\right).$$

For brevity, write $A_{d}=I_{p_{r}}\left(P,d+1\right)-p_{r}^{P}$, $B_{d}=I_{p_{r}}\left(P+1,d\right)-p_{r}^{P}I_{p_{r}}\left(1,d\right)$, and $C_{d}=I_{p_{r}}\left(P+2,d-1\right)-p_{r}^{P}I_{p_{r}}\left(2,d-1\right)$, so that

(A8) $$\mathrm{Pr}\left\{D_{r}\leq d\right\}=p_{r}^{P}+A_{d}q_{0}+B_{d}\left(q_{1}-q_{0}\right)+C_{d}\left(q_{2}-q_{1}\right),$$

and

(A9) $$E\left[D_{r}\right]=1-p_{r}^{P}+\sum_{d\geq 1}\left(1-p_{r}^{P}-A_{d}q_{0}-B_{d}\left(q_{1}-q_{0}\right)-C_{d}\left(q_{2}-q_{1}\right)\right).$$

We next analyze $-\sum_{d\geq 1}A_{d}$, $-\sum_{d\geq 1}B_{d}$ and $-\sum_{d\geq 1}C_{d}$, respectively. For $-\sum_{d\geq 1}A_{d}$, we have

(A10) $$\begin{array}{cc} -\sum_{d\geq 1}A_{d}= & \sum_{d\geq 1}\left(1-I_{p_{r}}\left(P,d+1\right)-\left(1-p_{r}^{P}\right)\right)\\ = & \sum_{d\geq 0}\left(1-I_{p_{r}}\left(P,d+1\right)\right)-\left(1-I_{p_{r}}\left(P,1\right)\right)-\sum_{d\geq 1}\left(1-p_{r}^{P}\right)\\ = & \frac{P}{p_{r}}-P-\sum_{d\geq 0}\left(1-p_{r}^{P}\right), \end{array}$$

where the last equality holds due to $I_{p_{r}}\left(P,1\right)=p_{r}^{P}$ and $\sum_{d\geq 0}\left(1-I_{p_{r}}\left(P,d+1\right)\right)=\frac{P}{p_{r}}-P$ by (5) and (6). For $-\sum_{d\geq 1}B_{d}$,

(A11) $$\begin{array}{cc} -\sum_{d\geq 1}B_{d}= & \sum_{d\geq 1}\left(\left(1-I_{p_{r}}\left(P+1,d\right)\right)-p_{r}^{P}\left(1-I_{p_{r}}\left(1,d\right)\right)-\left(1-p_{r}^{P}\right)\right)\\ = & \frac{P+1}{p_{r}}-\left(P+1\right)-p_{r}^{P}\left(\frac{1}{p_{r}}-1\right)-\sum_{d\geq 1}\left(1-p_{r}^{P}\right)\\ = & \frac{P}{p_{r}}-P+\frac{1-p_{r}^{P}}{p_{r}}-\sum_{d\geq 0}\left(1-p_{r}^{P}\right). \end{array}$$

For $-\sum_{d\geq 1}C_{d}$,

(A12) $$\begin{array}{cc} -\sum_{d\geq 1}C_{d}= & \sum_{d\geq 1}\left(\left(1-I_{p_{r}}\left(P+2,d-1\right)\right)-p_{r}^{P}\left(1-I_{p_{r}}\left(2,d-1\right)\right)-\left(1-p_{r}^{P}\right)\right)\\ = & 1-p_{r}^{P}+\sum_{d\geq 1}\left(\left(1-I_{p_{r}}\left(P+2,d\right)\right)-p_{r}^{P}\left(1-I_{p_{r}}\left(2,d\right)\right)-\left(1-p_{r}^{P}\right)\right)\\ = & 1-p_{r}^{P}+\frac{P+2}{p_{r}}-\left(P+2\right)-p_{r}^{P}\left(\frac{2}{p_{r}}-2\right)-\sum_{d\geq 1}\left(1-p_{r}^{P}\right)\\ = & \frac{P}{p_{r}}-P+\frac{2(1-p_{r}^{P})}{p_{r}}-\sum_{d\geq 0}\left(1-p_{r}^{P}\right). \end{array}$$

where the second equality holds due to $I_{p_{r}}\left(P+2,0\right)=I_{p_{r}}\left(2,0\right)=0$ by convention. By assumption, $q_{2}=1$. Thus, by plugging (A10), (A11), (A12) back to (A9), we obtain

(A13) $$E\left[D_{r}\right]=\frac{P}{p_{r}}-P+\frac{1-p_{r}^{P}}{p_{r}}\left(q_{1}-q_{0}\right)+\frac{2(1-p_{r})}{p_{r}}\left(q_{2}-q_{1}\right),$$

which directly implies (12) under the assumption that $q_{2}=1$.
